# Multiplex Surface-Enhanced Raman Scattering: An Emerging Tool for Multicomponent Detection of Food Contaminants

**DOI:** 10.3390/bios13020296

**Published:** 2023-02-19

**Authors:** Qingyi Wei, Qirong Dong, Hongbin Pu

**Affiliations:** 1 School of Food Science and Engineering, South China University of Technology, Guangzhou 510641, China; 2 Academy of Contemporary Food Engineering, South China University of Technology, Guangzhou Higher Education Mega Centre, Guangzhou 510006, China; 3 Engineering and Technological Research Centre of Guangdong Province on Intelligent Sensing and Process Control of Cold Chain Foods, Guangdong Province Engineering Laboratory for Intelligent Cold Chain Logistics Equipment for Agricultural Products, Guangzhou Higher Education Mega Centre, Guangzhou 510006, China

**Keywords:** multiple food contaminants, SERS, multicomponent detection, food safety, application

## Abstract

For survival and quality of human life, the search for better ways to ensure food safety is constant. However, food contaminants still threaten human health throughout the food chain. In particular, food systems are often polluted with multiple contaminants simultaneously, which can cause synergistic effects and greatly increase food toxicity. Therefore, the establishment of multiple food contaminant detection methods is significant in food safety control. The surface-enhanced Raman scattering (SERS) technique has emerged as a potent candidate for the detection of multicomponents simultaneously. The current review focuses on the SERS-based strategies in multicomponent detection, including the combination of chromatography methods, chemometrics, and microfluidic engineering with the SERS technique. Furthermore, recent applications of SERS in the detection of multiple foodborne bacteria, pesticides, veterinary drugs, food adulterants, mycotoxins and polycyclic aromatic hydrocarbons are summarized. Finally, challenges and future prospects for the SERS-based detection of multiple food contaminants are discussed to provide research orientation for further.

## 1. Introduction

Food contaminants such as foodborne microorganism, pesticides, and illegal additives can lead to biochemical detriments in human health [1]. On the basis of the World Health Organization’s statistical data, foodborne diseases currently affect nearly 10% people worldwide (https://www.who.int/zh/news/item/27-05-2022-seventy-fifth-world-health-assembly (accessed on 27 May 2022)). Alarmingly, contamination of multiple food contaminants has been increasingly reported. Furthermore, multiple food contaminants can exacerbate adverse effects on human health due to synergistic effects [2]. For example, over 40% grain samples collected from Asia, Europe, and America were contaminated with two or more mycotoxins [3]. Thus, it is of great importance in food safety to pay careful attention to multiple food contaminant detection.

To date, the most frequent utilized detection technologies, including high-performance liquid chromatography–mass spectrometry [4], electrochemical [5], fluorescence, and enzyme-linked immunosorbent assay (ELISA) [6], with superior selectivity and sensitivity, have realized varying degrees of commercial success. However, a series of conundrums including high cost, time taken, background interferences and signal overlap still hinder their application in the analysis of multiple food contaminants [7]. In addition, common rapid methods are good at detection of one target with high specificity, but there are more than one pesticides or veterinary drugs in food samples [8]. For example, electrochemical detection requires multiple electrodes or devices, which is costly and complicated for realizing multicomponent detection [9,10]. Additionally, broad fluorescence greatly inhibits its application in multicomponent detection [11]. Therefore, there is an increasing requirement of novel strategies for multiple food contaminant determination [12].

**Figure 1:**
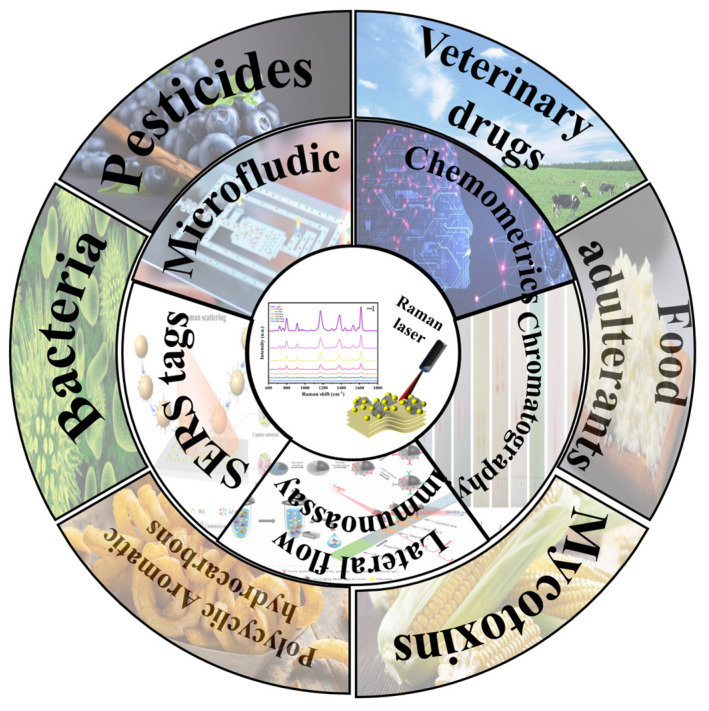
Illustration of SERS−based multicomponent detection in the food industry.

Raman spectroscopy is based on inelastic scattering effects, which can provide the structural and chemical information of components. Compared with other technologies, the Raman technique has the following advantages: low cost, non-destructive, ease of rapid detection, resistance to high moisture environments and narrow spectral widths [13]. However, the signal of Raman scattering is weak, and further application of Raman spectroscopy is limited [14]. Surface-enhanced Raman spectroscopy (SERS), integrated with nanotechnology and the Raman spectra technique, has emerged as an excellent detection technique. Superior to conventional Raman spectroscopy, the Raman signal of SERS is improved by approximately 6 fold, which facilitates the detection of trace contaminants. In addition, the acquired narrow fingerprint peaks make it possible to separate individual contaminants from a multicomponent system [15]. Thus, SERS has broader prospects over other competitors in the detection of multicomponents. As illustrated in Figure 1, boosted by the advancement of sensing technology, SERS has shown great potential in multiple food contaminant detection [16], such as foodborne microorganisms, drug residues, and illegal additives.

For food contaminant determination utilizing SERS, a few related reviews have been published in recent years. Zhang et al. [17] reviewed detection techniques based on magnetic SERS biosensors and summarized their sensing applications in detecting microorganism in the food industry. Additionally, Wu et al. [18] presented a review of the utilization of the SERS technique to detect mycotoxin contamination in agri-foods. Further, Zhou et al. [19] highlighted the SERS and its advanced compatible methods for determination of bacterial cells and bacterial metabolites in the food matrix. However, related reviews of multiple food contaminant simultaneous detection are scarcely reported. For example, Fan and co-workers [20] summarized the utilization of optical multiplexing bioassays to determine multiple biomarkers. Laing and colleagues [21] discussed the advances in SERS-based sensors for multiple cellular detection. Thus, it is necessary to supply a comprehensive explanation of the SERS-based multicomponent detection technique, clarifying the current applications of SERS in multiple food contaminant detection.

The current review aims to introduce the various strategies in terms of SERS techniques for multicomponent simultaneous detection followed by presenting an overview of their application in multiple food contaminant detection. Finally, the defects and prospects of SERS multicomponent detection will be listed. This review will provide a better understanding of the simultaneous detection of multicontaminants in food materials and products, and promote the adoption of SERS in food safety supervision.

## 2. SERS-Based Multicomponent Contaminant Detection Strategies

### 2.1. Multiplex SERS Enabled by Fingerprint

Normally, the Raman peaks of different functional groups are located at different Raman shifts, which provides a prerequisite for Raman spectroscopy to detect multicomponents [22]. Additionally, the narrow special peak widths make it easy for separating Raman spectra features among different components. Therefore, it is emphasized that SERS provides a convenient method for multicomponent determination in terms of the perspective of spectral characteristics [16]. For instance, Chen and co-workers [23] designed a flower-like molybdenum sulfide coated with silver nanoparticles (MoS_2_@Ag) to achieve SERS detection of multiple pesticides. Due to the narrow Raman peaks of tetramethylthiuram disulfide (TMTD) and methyl parathion (MP), the Raman peaks at 1376 and 1344 cm^−1^ could be clearly distinguished without any interference, which were ascribed to TMTD and MP, respectively.

However, SERS cannot provide accurate chemical information about multicomponents when the Raman characteristic peaks overlapped. Thus, efficient differentiation of spectra is a vital precondition for multicomponent detection. Additionally, chemometrics and separation techniques assisting SERS multicomponent detection strategies are introduced in the following section, respectively [24].

#### 2.1.1. Multicomponent Detection by SERS Assisted with Chemometrics

Generally, the SERS detection process will generate vast spectral data, and the phenomenon of spectral overlap is inevitable in multicomponent detection. It is unsuitable for the analyst to detect the multicomponent contaminants only using the SERS technique. Therefore, spectra pretreatment methods become particularly significant for multicomponent detection. Chemometrics are proposed to assist SERS for matrix overlapping spectra processing, which is able to automatically classify and separate spectra or data [25]. In many circumstances, the chemometrics can be divided into unsupervised and supervised pattern recognition according to whether there is a database prepared in advance [26,27,28].

**Figure 2:**
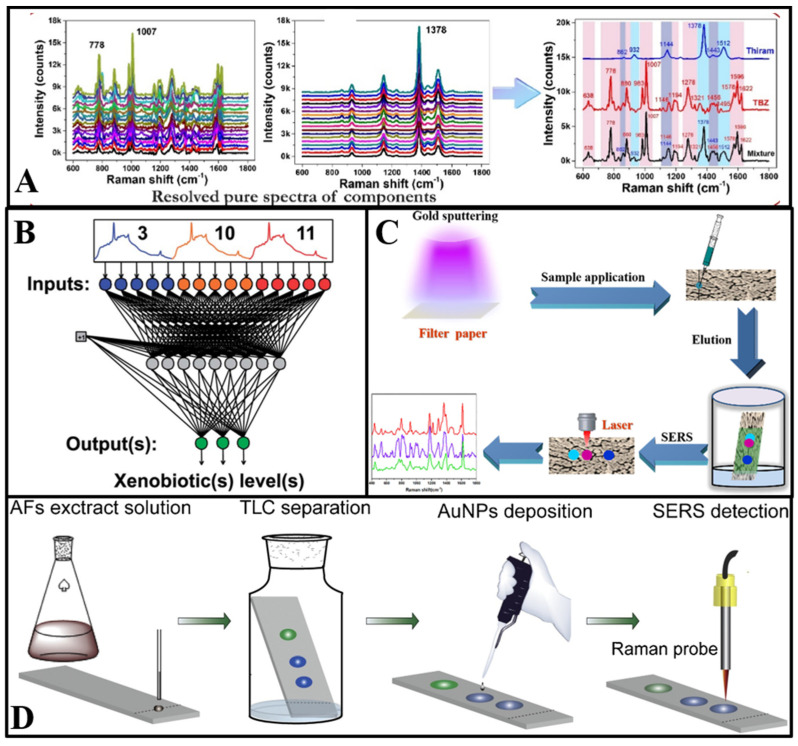
(**A**) Schematic diagram of the process and outcome of a self-modelling mixture analysis of the SERS spectra of pesticides mixture [34]. (**B**) Schematic structure of a neural network with 15 input layers and 3 output layers [40]. (**C**) Illustration of the Au−PC strips and combination of PC separation and SERS detection for multicomponents [41]. (**D**) Schematic illustration of on−site AFs detection in water using TLC−SERS [42].

Unsupervised pattern recognition is defined as machine learning without a training set, which can visualize dissimilarity among multicomponents via artificially reducing the spectral data dimension [29]. Among the most used unsupervised pattern recognition methods, principal component analysis (PCA) has been widely used to classify SERS spectra data of multicomponents [30]. The dimension reduction procedure of principal components (PCs) enables a PCA score plot in the dimension reduction space, and realizes the purpose of multicomponent visual classification [31]. In the case of multicomponent detection, the points on the score plot represent the relationship between the Raman shifts of the main SERS peaks and different target analytes [32]. Thus, the PCA method can effectively avoid the interference of miscellaneous peaks in Raman spectra, and further improve the reliability of SERS-based multicomponent detection. Furthermore, self-modelling mixture analysis (SMA) is another useful chemometric established on the alternating least squares approach, proposed to extract the pure spectra of single components from the mixed SERS spectra of multicomponents [33]. As shown in Figure 2A, with the help of self-modelling mixture analysis, the Raman spectrum of multiple pesticides can be disassembled to pure spectra of each pesticide, and the simultaneous determination of multiple pesticides with overlapped SERS spectra was realized [34]. This method effectively solves the following problems: (i) extraction of the SERS signals for pure contaminants from mixed spectra where the Raman peaks are overlapping; (ii) quantitation and detection of multiple food contaminants simultaneously without any sample pre-treatment.

Supervised pattern recognition is another method for classification and quantitative prediction of multicomponent mixed Raman spectra [31]. Different from unsupervised pattern recognition, supervised pattern recognition has advantages in the classification of unknown samples, enabling detection of multiple targets [29]. Partial least- squares regression (PLSR) is the most used supervised pattern recognition for classification and quantitative prediction of multicomponent spectral data [35]. Additionally, PLSR can effectively process SERS data of multicomponents, especially if the Raman signals of single component are weak, or the concentration of single components is relatively low in multicomponents [36]. As another supervised pattern recognition method, the artificial neural network (ANN) is defined as a structure comprised of densely interconnected adaptive simple processing elements that is capable of performing massively parallel computations for data processing and knowledge representation [29]. Typically, an artificial neural network consists of an input layer providing data of the multicomponent Raman spectrum, a hidden layer loaded with the training system, and an output layer (Figure 2B). Normally, the number of neurons in the output layer determines the quantity of target components [37]. The utilization of an artificial neural network can provide significant improvements for the multicomponent analytical performance of SERS. Among the major advantages of an ANN is that it can be applied when the number of multicomponents is large [38]. As the extension of the neural network, deep learning, including the convolutional neural network (CNN), restricted Boltzmann machine (RBN), deep belief networks (DBN), stacked auto-encoders, has become a hot research topic, and it is sought after in numerous fields. Combining deep learning with the SERS technique enables further enhancing the classification ability of multiple SERS data. For example, Li and co-workers [39] utilized the Resnet-based deep learning model to classify six kinds of organophosphorus pesticides simultaneously. The results indicated that identification accuracy could exceed 92% and the whole SERS multicomponent detection process could be completed within 20 min.

Accordingly, recent years have witnessed a booming applicability of chemometrics in SERS detection. As a rapid detection technology, SERS assisted with chemometrics can achieve a majority of multicomponent detection. Standing in the era of big data, chemometrics must progress with the development of the technique, and SERS-based multicomponent detection is also bound to change with each passing day. However, there is a part of multicomponent spectral data that cannot be classified or quantified by chemometrics simultaneously. 

#### 2.1.2. The Paper Chromatography-Based SERS Technique for Multicomponent Detection

In order to realize simultaneous detection of multicomponents, several other separation technologies are proposed to be combined with SERS, such as paper chromatography and thin-layer chromatography. Paper chromatography (PC) is a conventional partition chromatography based on the capillary forces, and such a technique can separate multicomponents according to different partition coefficients [43]. However, the lack of accuracy and sensitivity of paper chromatography have limited its application in high-performance detection. Fortunately, due to the development of the surface-enhanced Raman scattering-based spectroscopic technique, paper chromatography has drawn some attention back to the field of quantitative detection, especially for multicomponent detection.

The utilization of PC technology can complement the drawbacks of SERS in multicomponent detection [43]. Song et al. [41] proposed a filter paper sputtered with gold nanoparticles (AuNPs) as the SERS substrate for multicomponent detection. Firstly, the paper substrate was dripped with a solution of three kinds of antifungal agents. Then, the antifungal agents migrated to different spots in the paper chromatography strips owing to their different chemical structure and corresponding adsorption–desorption behavior. Additionally, the spectra of malachite green, crystal violet, and methylene blue in different spots could be recorded through the Raman laser (Figure 2C). The combination of SERS and the paper chromatography technique showed the feasibility and trace analytical performance of multicomponent detection. Furthermore, in Jin et al.’s [22] study, a PC–SERS device modified by silver nanoparticles and zinc oxide nanoparticles was developed to detect thiuram and dimethoate simultaneously. With different diffusion coefficients, thiuram and dimethoate were separated to different spots. Additionally, the SERS spectra were measured after complete diffusion.

Generally, the PC–SERS platform has great detection performance, and is simple, portable, and sensitive [43]. As a joint technology, the PC–SERS device integrates separation, capture and detection abilities, which undoubtedly has a broad application. However, the migration distance is limited by the adsorption capacity of target analytes, causing analyte coverage of the paper substrate [44]. The paper chromatography–SERS device still has much space for further development in future explorations, which may inspire more innovative applications for multicomponent detection.

#### 2.1.3. The Thin-Layer Chromatography-Based SERS Technique for Multicomponent Detection

To further improve separation ability and meet the requirements of SERS-based multicomponent detection, another chromatographic technology named thin-layer chromatography (TLC) was introduced. Distinguished from the separation capability of paper chromatography, the TLC technique allows separating the target analytes with weak adsorption [24]. With reliable separation performance and stable properties, the integration platform of thin-layer chromatography and the SERS technique (TLC–SERS) can be used to separate and detect multicomponents simultaneously. 

As shown in Figure 2D, the TLC plate deposited with AuNPs was designed as a substrate at first. Additionally, aflatoxins dissolved in ethanol were immobilized on the bottom of the TLC plate. Then, a solvent consisting of acetone and chloroform was added to the plate and migrated through the gel layer via capillary action. Aflatoxins with different affinities were separated to different points as the solvent migrated. The characteristic peaks could be detected through laser irradiation at different sample points [42]. Furthermore, the TLC–SERS device has potential application for target analytes with similar chemical structures [45]. Cai et al. [46] used the TLC–SERS device to determine benzidine and 4-aminobiphenyl in food contact material, with Raman peaks that are overlapped at 1614 cm^−1^. In their research, petroleum ether and ethyl acetate were used as eluents to reach optimal separation capability. Additionally, benzidine and 4-aminobiphenyl were quantitatively detected by on-plate SERS detection after separation. Meanwhile, Li et al. [47] established the TLC–SERS method for simultaneous detection of 14 citrus flavonoids. 

As aforementioned, the TLC–SERS device can be used to sensitively and accurately identify multicomponents in complicated systems, especially target analytes with overlapping Raman characteristic peaks. The TLC–SERS technology can play a vital role in fast screening of food contaminants by the combination of separation technologies and surface-enhanced Raman spectroscopy, which can eliminate the interference between different components, and enhance the multifunctionality of SERS detection.

### 2.2. Multiplex SERS Enabled by SERS Tags

Although Raman fingerprint-based multicomponent detection has its own advantages, it is limited to determine analytes without obvious Raman characteristic peaks [21]. In order to solve above-mentioned problem, researchers introduced SERS tags to determine multicomponents indirectly. The most common SERS tags are mainly composed of a Raman reporter, a capture probe (antibody or aptamer) and SERS enhancement nanoparticles, which exhibits multiabilities including recognition, capture and characteristic Raman signals generation [48]. SERS tags allows Raman signal variations arising from the presence or absence of detection targets. According to the difference in biological properties, SERS tags can be divided into two types, immunoassays and aptamer sensors [49].

#### 2.2.1. Multiplex Immunoassay 

Immune detection with antibody-decorated SERS tags has attracted increasing attention in food contaminant control, with which targets involving bacteria, mycotoxin, and other contaminants without SERS signals can be specifically detected and captured from solutions [50]. In general, the antibodies are bound to nanoparticles as capture probes through a group of chemical bonds such as Au-S, Au-N, and Ag-S [51]. It is noteworthy that antibodies cannot produce Raman signals. The Raman signals of SERS tags are produced by combining Raman reporters, and thio-small molecular, nitrogen cations or sulfur dyes are the most commonly used as Raman reporters [52]. Due to the biocompatibility and chemical distinctiveness of Raman reporters for the analysis of multicomponents, as well as the advantages of SERS tags for multiplexed analysis, an immunoassay is a suitable candidate for multicomponent detection.

**Figure 3:**
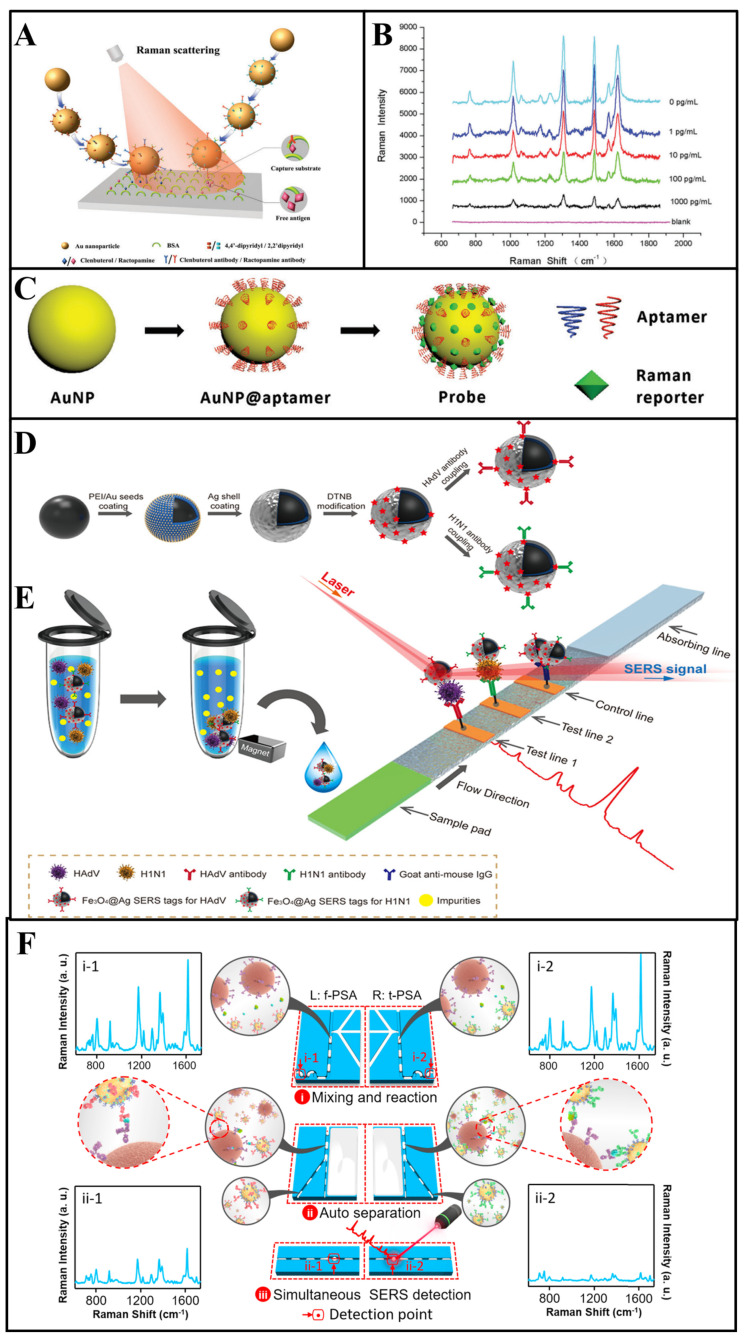
(**A**,**B**) Multiantibodies modified with gold nanoparticles for SERS multicomponent detection, and SERS spectra acquired with different clenbuterol and ractopamine concentrations [53]. (**C**) The fabricate of the aptamer-modified probe [56]. (**D**,**E**) Schematic diagram of the SERS strip for detecting viruses [57]. (**F**) Schematic design of the parallel microdroplet channels for simultaneous detection of f−PSA and t−PSA [58].

As an example, in the work by Yu et al. [53], a multiplex immunoassay based on competitive SERS was designed to detect clenbuterol and ractopamine simultaneously. As shown in Figure 3A, clenbuterol and ractopamine antibodies were tightly anchored on different gold nanoparticles through the Au-thiol bonds, and 4, 4′-dipyridyl and 2, 2′-dipyridyl, with different Raman peaks, were selected as Raman reporters to bind to different nanoparticles. The determination of multiantigens was achieved by competitive binding between target antigens and antigen–bovine serum albumin (BSA) immobilized on the capture substrates. As shown in the blue line in Figure 3B, in the absence of antigens, the SERS tags would combine with capture substrates modified with clenbuterol-BSA and ractopamine-BSA, and the Raman peaks of SERS tags would appear at 1612 and 1478 cm^−1^, respectively. Then, because of the binding of SERS tags with free antigens, the SERS tags captured by substrates gradually decreased with the increase in the free antigen concentration. During such multicomponent detection, SERS signals show a negative relationship with the concentration of target analytes. In another study, Bai et al. [54] used magnetic beads modified with *Escherichia coli* and *Staphylococcus aureus* antibodies to capture and separate bacteria from a complex solution, and two kinds of gold nanoparticles bound with Raman reporters were designed as the SERS tags to detect different bacteria simultaneously. In their study, the concentration of two bacteria is proportional to the signal intensity of two SERS tags. Normally, the feasibility of SERS tag-based multicomponent detection is largely determined by the selection of appropriate Raman reporters [55]. 

Owing to its mature fabrication techniques, antibody-modified SERS tags are widely utilized as attractive recognition elements in SERS multicomponent detection. In addition, SERS tags with signals in the Raman silent region can further avoid the phenomenon of spectral overlap, which enhances the accuracy of immunoassays for multicomponent detection simultaneously [59]. However, antibodies are expensive and unstable, and are inactivated in extreme environments [60]. To overcome these defects, aptamers have emerged as a robust alternative [54,56,61].

#### 2.2.2. Multiplex Aptamer Sensors

Aptamer sensors, as a kind of artificial oligonucleotide sequences, can specifically bind to target analytes with high binding accuracy, efficiency, affinity and good biocompatibility [51,62]. Furthermore, aptamers can be used as an alternative to antibodies to detect target analytes in complex environments. In recent years, aptamers have attracted the attention of a large number of researchers as they are highly robust, and have high affinity and chemical resistance [63]. Normally, aptamers can be modified onto the nanoparticle surface through Au-thiol bonds (Figure 3C). Nowadays, boosted by the continuous advancement of modification technology, aptamers have shown intrinsic value in the modification of SERS tags for multicomponent simultaneous detection [64]. 

Zhang et al. [65] proposed vancomycin-modified Fe_3_O_4_@Au magnetic nanoparticles (Fe_3_O_4_@Au MNPs) to detect and capture *Escherichia coli* and *Staphylococcus aureus* simultaneously. Additionally, two batch gold nanoparticles were modified with different Raman reporters and aptamers as the tags, respectively. In the presence of the target analytes, Fe_3_O_4_@Au MNPs capture bacteria via hydrogen bonding between the bacterial cell wall and vancomycin at first. Then, the SERS tags bind to Fe_3_O_4_@Au MNPs through the binding of aptamers and antigens. Finally, the sandwich structures were separated by a magnetic force. The concentration of the two bacteria is determined via observing the enhancement of Raman signals at 1331 and 1074 cm^−1^.

Multiplex aptamer SERS tags can be used to detect multicomponents indirectly, and the Raman signal transformation is influenced by the binding mode of Raman reporters with nanoparticles [66,67]. However, these SERS tag-based multicomponent detection strategies are also susceptible to the position of characteristic peaks of Raman reporters, which have great boundedness in distinguishing fingerprints.

#### 2.2.3. Multichannel Lateral Flow Immunoassay

Based on SERS tags, a powerful immunoassay technique named lateral flow immunoassay (LFIA) is established, which is a point-of-care bioassay performed on a paper-based device [68]. A typical LFIA strip consists of a sample pad and a conjugation pad, which can determine analytes via fabricating an immunocomplex of antibody and antigen on the test line. Such LFIA strips allow the simultaneous determination of multicomponents while multiple test lines are set. As aforementioned, the LFIA technique is a novel idea for SERS-based multicomponent detection, and SERS-based LFIA (LFIA–SERS) platforms have been widely researched in recent years [69]. In SERS–LFIA platforms, different antibodies are modified on multiple test lines, which can capture the SERS tag–target complex in solution. Additionally, different SERS signals could be determined in test lines, respectively. Due to the specific separation capacity of LFIA strips for the multicomponents, as well as the advantages of SERS tags for multiplexed analysis, the LFIA–SERS technique is a suitable candidate for multicomponent detection.

For example, in the work by Wang et al. [57], two kinds of Raman reporters labelled silver-coated Fe_3_O_4_ magnetic nanoparticles (Fe_3_O_4_@Ag MNPs) were modified with influenza A H1N1 virus and human adenovirus antibodies as the SERS tags (Figure 3D). With the presence of the target analytes, the SERS tags could form “antigen–SERS tag” immunocomplexes, which would separate from the solution by a magnetic force in the next step. Subsequently, two kinds of analyte–SERS tag complexes were captured by different antibodies that immobilized on different test lines, respectively (Figure 3E). For the SERS signal readout, a completed LFIA strip was scanned by a Raman laser in a Raman microscope system for the determination, where the SERS peaks intensity of different test lines were recorded for multicomponent detection. Using the separation ability of LFIA, multicomponent determination using SERS is realized without overlap of characteristic peaks [70]. Furthermore, Wang and co-workers [71] developed GO@Au/Ag SERS tags modified with four kinds of antibodies, which can capture target bacteria rapidly. Additionally, the different “SERS tag–bacteria” structures could form “SERS tag–bacteria–antibody” sandwich conjugates in two test lines. The SERS intensity of two test lines was determined for quantitative analysis of four bacteria simultaneously.

Recent analysis showed that the LFIA–SERS method can prevent the interference of the competitiveness between different components [72,73,74]. In an ideal LFIA–SERS device, multicomponents can be separated to different test lines evenly. In this case, the theoretical LODs of different components is equal to the actual concentration [75]. Although LFIA–SERS method-based multicomponent detection has great potential, SERS tags are expensive and such platforms cannot be reused, which greatly increases the detection cost and inhibits its practical application. Thus, while designing a reasonable multicomponent LFIA–SERS platform, the corresponding economic benefits should also be considered.

#### 2.2.4. Multichannel Microfluidic

In order to enable SERS for multicomponent detection in a more controlled system, it is necessary to develop a stable and reliable technology to combine with SERS. The development of a microfluidic system has recently gained significant attention from scientific communities. Microfluidic chips extend chemical reactions, physical reactions or biological reactions to the micron scale via chip manufacture by the microelectromechanical system [76]. Furthermore, the microfluidic chip can also be designed as a multichannel or multilayer structure to realize the objective of multicomponent simultaneous detection. With the support of syringe pump pressure, the fluid will be pushed to different channels and captured by SERS tags that are installed in parallel-segmented chambers. Additionally, the target components can be simultaneously identified by Raman spectra [77]. Thus, SERS–microfluidic platforms with different selective determination capability are still a long-awaited requirement, which can be beneficial for multicomponent simultaneous detection in practical applications.

For example, Yang and colleagues [78] proposed a SERS–microfluidic system with multiple gas sensor detection to determine nine different gases simultaneously. The microfluidic chip was composed of three different detection units—U_A_ (MXene/Au), U_B_ (MXene/Au/2,4-dinitrophenylhydrazine (DNPH)) and U_C_ (Ag@Raman reporter). To be specific, UA could capture aromatic gases via physisorption, and SERS spectra could directly detect the signal peaks of aromatics; UB detected ketones and aldehydes through linker-mediated chemisorption, which could be analyzed by SERS peaks of the reaction products of the DNPH with target gases; UC could detect hydrogen sulfide (H_2_S) gases via the construction of Ag-S bonds, which could effectively improve the signal intensity of the Raman reporter. Thus, the simultaneous determination of multicomponents could be realized in a single chip without a separation technique, which would greatly improve the convenience of analysis. Furthermore, for the microfluidic-based SERS device, the design of a channel and a chamber can also realize SERS multicomponent detection. In another case, to simplify the operation procedure of multicomponent detection, Gao et al. [58] designed a microfluidic-based SERS device with two parallel channels, and completed the simultaneous detection of two kinds of prostate-specific antigens (f-PSA and t-PSA). In the presence of antigens, two kinds of SERS tag–antigens composite were formed in two parallel channels and the strong SERS intensities could observed in Figure 3F (i-1 and i-2). Then, the separation of pure SERS tags and SERS tag—antigen composites were achieved via the formation of “SERS tag–antigen–magnetic bead” sandwich structures. Thus, as shown in ii-1 and ii-2, the SERS signals decreased significantly.

To summarize, with reliable performance and a diversified design, the integrated SERS–microfluidic platforms exhibit an effective strategy for multicomponent detection. The integrated platforms have a short analytical time, multicomponent screening capability, fewer interferences, and good stability, which pave the way for the commercial adoption of SERS for multicomponent simultaneous determination.

## 3. Application of SERS in Multifood Contamination Determination

Food contamination, consisting of abiotic (including pesticides, veterinary drugs and illegal additives, heavy metal, polycyclic aromatic hydrocarbons, and polychlorinated biphenyls) and biotic contamination (bacteria, mycotoxin and foodborne viruses), can greatly influence food safety [2]. Food contamination is universal, with the severest form being multiple food contaminants coexisting in food, which will cause more serious harm to humans. Thereby, to ensure food safety, modern detection technologies should include detection of multicomponents simultaneously [79]. As illustrated above, SERS is a powerful technology for multicomponent detection. Capture technologies, separation technologies and chemometric methods play major roles in promoting multicomponent detection. The applications of SERS in multiple food contaminants are discussed in detail. And the Table 1 summarizes recent applications of SERS detection in multifood contaminants.

### 3.1. Determination of Multi-Bacteria 

Foodborne bacteria are commonly considered as among the major causes of food-associated disease around the world. With the development of food logistics, a complicating issue is that there may be more than one species of bacteria in the food sample, and the simultaneous spread of multifoodborne bacteria has remarkably increased and is a threat to human life [100]. Thereby, rigorous detection of multifoodborne bacteria in agriculture and animal husbandry products is of great importance in protecting public health [101].

**Figure 4:**
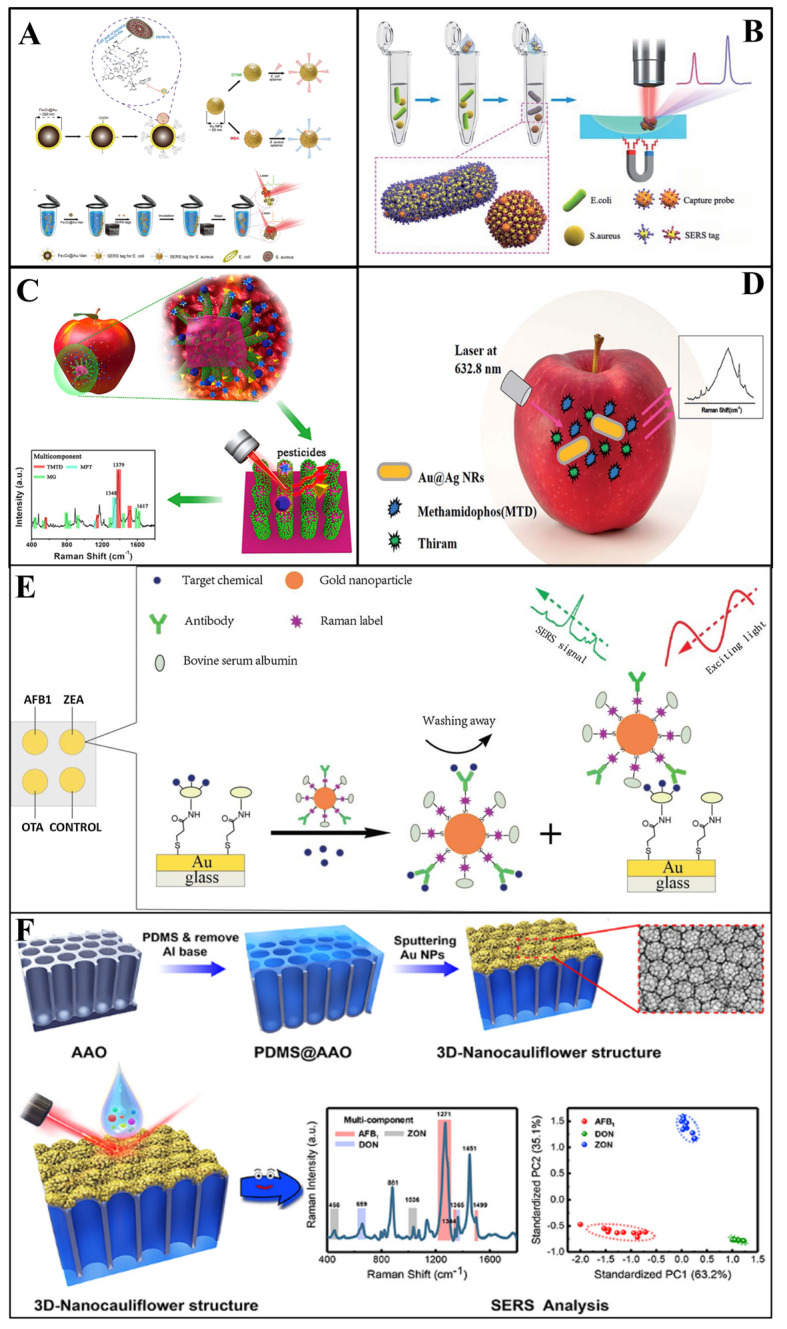
(**A**) Schematic illustration of the synthesis of a vancomycin−modified SERS platform [65]. (**B**) Schematic illustration of the SERS−based sandwich immunoassay platform [54]. (**C**) Schematic demonstration of the preparation of a G–SERS substrate [84]. (**D**) Schematic illustration of the simultaneous detection of thiram and methamidophos [85]. (**E**) Schematic demonstration of a multiplex SERS−based immunosensor [98]. (**F**) Schematic demonstration of the 3D nanocauliflower substrate [99].

In Duan et al.’s [67] study, a polydimethylsiloxane (PDMS) film was modified with gold nanoparticles and bacteria aptamers as SERS substrates, then the gold nanoparticles modified with aptamers were fabricated with Raman reporters to act as SERS tags. *Vibrio parahaemolyticus* and *Salmonella typhimurium* in seafood samples were simultaneously captured and detected by this sandwich structure, with an LOD of 18 and 27 cfu/mL, respectively. Similarly, Zhang et al. [65] used this sandwich structure to determine *Escherichia coli* (*E.coli*) and *Staphylococcus aureus* (*S. aureus*) simultaneously, which were detected at sconcentration as low as 20 and 50 cfu/mL, respectively (Figure 4A). Furthermore, Shen and colleagues [102] proposed an immunochromatographic assay (ICA) modified with Au shell-coated graphene oxide nanosheets as SERS substrates. As a result, the simultaneous determination of *E.coli*, *S. aureus* and *Salmonella typhimurium* in skim milk could be achieved, with a LOD of 10, 10, and 8 cfu/ mL. Recently, Li and co-workers [103] developed a SERS (Fe_3_O_4_@Au)-based ICA for the simultaneous detection of *Salmonella typhimurium* and *S. aureus* in milk. The LOD values for *Salmonella typhimurium* could reach 12 and 9 cfu mL^−1^, respectively. In 2020, in order to achieve higher sensitivity, Bai et al. [54] addressed the rapid detection of *E.coli* and *S. aureus* in water and milk by developing a SERS method based on antibody-modified tags. The LOD for *E.coli* and *S.aureus* was as low as 10 and 25 cfu/mL, respectively (Figure 4B). The feasibility of SERS in multifoodborne bacteria detection was also proved in Wu [73] and Meng et al.’s [81] experiments. 

### 3.2. Determination of Multi-Pesticides

Pesticides are an indispensable part of modern agriculture, and make outstanding contribution to protecting the effective growth and reproduction of crops and vegetables. However, due to abuse and overuse, most use of pesticides eventually results in residues that threaten human health [104]. Additionally, the simultaneous utilization of various pesticides in agricultural activity has caused a great challenge to pesticide monitoring. Fortunately, with the development of the SERS technique, many SERS-based multicomponent detection methods have been proposed for multiple pesticide detection simultaneously [105]. A brief discussion of multiple pesticide determination is given in this section.

Zhang et al. [85] used silver-coated gold core-shell nanorods as SERS substrates to conduct SERS for the detection of two kinds of pesticides (thiram and methamidophos) in the apple surface (Figure 4C). In their study, the extraction was completed by ethanol dropped onto the apple surface with pesticide residues. Additionally, the results demonstrated that SERS can achieve the simultaneous detection of multi-pesticides, and the LOD of thiram and methamidophos was 4.6 × 10^−7^ M and 4.4 × 10^−4^ M, respectively. In addition, Wang et al. [86] successfully detected a mixture of acephate, cypermethrin and tsumacide using the SERS spectrum from apple peels (Figure 4D). The mixture spectrum demonstrated the high level of sensitivity of the proposed SERS method with LODs of 10^−3^, 10^−3^ and 10^−4^ ng/cm^2^, respectively. Furthermore, novel Ag NWs@ZIF-8 core-shell nanochains were proposed for the simultaneous detection of MP and carbaryl (CBL) in cabbage by Yang and co-workers [106]. In their research, the as-prepared substrates achieved the sensitive determination of MP and CBL, with LODs as low as 7.6 × 10^−9^ and 5.7 × 10^−9^ M, respectively.

### 3.3. Determination of Multi-Veterinary Drugs 

Veterinary drugs are an integral part of animal husbandry and are widely used for preventing, treating, or diagnosing animal diseases. In order to achieve efficient antibacterial effect and disinfection, it is common to use multiple veterinary drugs together in the livestock, poultry and aquaculture industry, which leads to great trouble in detection [107]. Similar to pesticides, with the increasing amounts of veterinary drugs employed in animal husbandry, drug residues lead to ineluctable pollution in animal products. Thus, it is necessary to introduce the SERS technique to achieve the simultaneous identification of multiple veterinary drugs.

Nitroimidazoles are a group of veterinary drugs consisting of a 5-nitroimidazole ring structure that has been proven to be mutagenic and potentially carcinogenic. Combing the high separation of the TLC technique and the SERS performance of AuNPs, Shi’s group [108] reported a TLC–SERS strategy to detect 14 nitroimidazole compounds in actual pork samples, reaching a LOD of 0.1 mg/L. In addition, a group of veterinary drugs known as sulfonamides are a common public threat. Lai et al. [94] demonstrated that the simultaneous detection of a group of sulfonamide residues in meat by SERS was feasible. With the assistance of principal component analysis, sulfamerazine, sulfamethazine and sulfamethoxazole drugs in pork could be detected at concentration levels as low as 10 ng/mL^−1^. In another study, Chen et al. [95] evaluated the use of gold nanorods substrates to detect malachite green and crystal violet in fish simultaneously. They found that the determination limit could decrease to 1 ppb for both malachite green and crystal violet. Furthermore, Shan and co-workers [109] constructed a AgNPs@Si SERS substrate for simultaneous analysis of enrofloxacin and ciprofloxacin hydrochloride monohydrate in water. As a result, a minimal detection of 10^−10^ and 10^−8^ M for enrofloxacin and ciprofloxacin hydrochloride monohydrate could be realized, respectively. 

In summary, SERS platforms showed potential for simultaneously monitoring multiple veterinary drugs, paving the way for a low-cost and time-saving strategy to monitor and prevent the dangers of veterinary drug residues.

### 3.4. Determination of Multifood Adulterants 

Food adulterants are illegal materials that are used to replace the original nutrients to satisfy industry standards and seek economic benefits, and cause irreversible serious hazards to consumers [110]. There is a long history associated with foodborne disease outbreaks due to the abuse of multiple food adulterants, and our planet is at the precipice of a huge global additive safety concern. It is necessary to develop a novel strategy which is rapid and highly sensitive in the simultaneous detection of multiple illegal additives in food. In recent years, SERS-based technologies are proposed for multiplex detection of food adulteration.

As a typical example, melamine and thiocyanate ion (SCN-) are the most illegal food adulterants in milk powder, affecting a whole generation of babies. Yang et al. [96] developed a SERS detection method based on sliver nanoparticles for the detection of thiocyanate ion and melamine in milk. In their experiment, sodium chloride and sodium hydroxide were used as the aggregating agent and the alkaline adjusting agent, respectively. Through research, it was found that the linear ranges are 2.00–191.00 mL/L and 0.01–4.80 mg/L for thiocyanate ion and melamine, respectively. In addition, Li and colleagues [111] constructed hemp spherical AgNPs as a SERS platform for the SERS determination of sunset yellow, lemon yellow, carmine and erythrosine adulteration in black tea. The strategy could detect the above-mentioned adulterants down to 0.1 μg/mL. In another study, Li et al. [98] had similar success in the simultaneous detection of six glucocorticoids (prednisone, prednisone acetate, prednisolone, hydrocortisone, hydrocortisone acetate, and dexamethasone) that are illegally added in dietary supplements by a TLC–SERS device. The results indicated that spot concentrated Raman scattering combined with TLC could achieve a greater level of accuracy, with LODs of 4, 4, 4, 6, 6, and 4 µg, respectively.

### 3.5. Determination of Multi-Mycotoxins

Mycotoxin is a type of contaminant in food that is metabolized by fungi during harvest, storage, and processing. The existence of mycotoxins in foodstuff has been considered a potential hazard to human health. Moreover, recent studies have demonstrated that the coexistence of mycotoxins can pose more serious effects on people [99]. Therefore, the development of the multiple mycotoxin detection technique is a significant matter for the microbial safety of food. 

As two typical mycotoxins, deoxynivalenol (DON) and trichothecenes (T-2) are generated by Fusarium graminearum with a similar structure. In general, the simultaneous determination of these significant is very challenging. Ge et al. [112] fabricated a MOF-74 (Ni)-based SERS substrate (NiRs@MOF-74 (Ni)/Ag) for detection and analysis of DON and T-2 simultaneously. The NiRs@MOF-74 (Ni)/Ag substrate featured high sensitivity and an enrichment effect of magnetic NiRs, adsorption of MOF-74 (Ni) and localized surface plasmon resonance properties of AgNPs, enabling a low LOD (0.08 and 0.15 μg/L) for the determination of DON and T-2. Furthermore, aflatoxins are frequently found in crops, such as peanuts, wheat and soybeans. Considering their hazard to human, Qu et al. [42] simultaneously determined four aflatoxins (AFB_1_, AFB_2_, AFG_1_, and AFG_2_) by using the TLC-SERS device. The quantification results illustrated that the limit of detection was 1.5 × 10^−6^, 1.1 × 10^−5^, 1.2 × 10^−6^, and 6.0 × 10^−7^ M for four aflatoxins, respectively. For detection of other mycotoxins, there are also reports in the research of Li et al. [98]. In their study, aflatoxin B1, zearalenone, and ochratoxin A in corn and wheat were simultaneously determined by using a SERS-based immunosensor platform (Figure 4E). As a result, the limit of detection was 0.061–0.066, 0.53–0.57 and 0.26–0.29 μg /kg, respectively, and indicated the sensitively and rapid detection capability of SERS for multi-mycotoxin detection. In another study, Li et al. [99] detected aflatoxin B1, deoxynivalenol, and zearalenone in maize, using SERS based on a cauliflower-inspired 3D SERS substrate (Figure 4F). The LOD reached as low as 1.8, 47.7, and 24.8 ng/mL, respectively. In summary, the development of surface-enhanced Raman spectroscopy can promote the simultaneous determination of mycotoxins as well as efficiently avoiding foodborne disease.

### 3.6. Determination of Multi-Polycyclic Aromatic Hydrocarbons 

Polycyclic aromatic hydrocarbons (PAHs) consist of fused aromatic rings without any replication groups. PAHs are among the main food contaminants in the processing and cooking of food at high temperatures. They are of great concern for human health because of their accumulation, migration, and transformation in the food chain [113]. SERS has been widely used in the detection of many PAHs.

For instance, Qu et al. [114] used SERS coupled with silver nanoparticles to simultaneously determine a group of PAHs, including anthracene, fluoranthene, pyrene, and 3,4-benzopyrene. By analysis of the mixture spectrum, they could classify four PAHs from different characteristic peaks, with a limit of detection of 1.2 × 10^−5^, 0.8 × 10^−5^, 1.3 × 10^−5^, and 0.9 × 10^−5^ M, respectively. Furthermore, Wang et al. [115] also used SERS coupled with Au nanoparticles and reoxidized graphene oxide to simultaneously detect 16 kinds of PAHs in Chinese traditional fried food. By infusing binary linear regression to the data analysis, they could classify a mixture of 16 kinds of PAHs, with a linear correlation coefficient ranging from 0.9889 to 0.9997. In another study, benzo[a]pyrene and anthracene were simultaneously determined by Renard et al. [116]; they used the polymer polydopamine to functionalize the aluminum nanocrystals, and this substrate can capture polycyclic aromatic hydrocarbon pollutants in water. As a result, an LOD of approximately 2.11 nM for both PAHs was reached. Overall, these applications depict the usability of SERS-based multicomponent detection techniques for detecting multi-polycyclic aromatic hydrocarbons. 

### 3.7. Determination of Other Multifood Contaminants

In addition to the above applications of multicomponent detection, SERS technology is also often used in other multifood contaminant detection [117,118]. For example, Song et al. [41] have simultaneously detected chromium, copper and nickel ions in rice through the gold nanoparticles modified by the Raman reporter (4-MBA). In the presence of the target heavy metal ions, the Raman reporter was connected to gold nanoparticles and produced intense Raman signals. The strategy was successfully exploited for simultaneous detection of three heavy metal ions at 10^−7^ M. Furthermore, Tian and co-workers [119] designed a new SERS aptasensor for simultaneous detection of Hg^2+^ and Ag^+^ in water. As a result, multiple detection method of the SERS aptasensor could avoid the interference between different metal ions and realized a lower LOD of 4.40 and 9.97 am for Hg^2+^ and Ag^+^, respectively. In addition, Neng et al. [120] used SERS coupled with a robust immunoassay using silica-encapsulated antibodies and modified SERS nanotags to simultaneously determine the west Nile virus, the rift valley fever virus, and Yersinia pestis in bovine serum. As a result, the limit of detection of the three virus is 10 pg/mL, 100-fold lower than with the antigen capture assay. In addition, Sanchez-Purra et al. [121] used the LFIA–SERS device to detect the Zika and dengue viruses simultaneously, and the detection limit of the SERS method was 15-fold and 7-fold lower than for the dipstick immunoassay. In addition to multifoodborne virus detection, Dhakal et al. [33] proposed self-modelling mixture analysis method-based SERS technology to determine the Sudan-I and metanil yellow pixels in curry powder simultaneously. All the above studies demonstrate the wide practicability of SERS technology in multifood contaminant detection.

## 4. Outlook and Conclusions

In the current review, SERS-based multicomponent detection strategies and their applications in multiple food contaminant simultaneous detection are elaborated. From fingerprint-based multicomponent detection, multiple SERS data processing assisted with chemometrics, and separation–detection integrated platforms to the SERS tag-based multicomponent detection technique and beyond, SERS provides a variety of advanced technologies to satisfy basic goals in multiple food contaminant determination. However, troubles and challenges still exist in practical application, and several fields require further research.

(1) Raman spectral fingerprint-based multicomponent detection is not always effective due to overlapped Raman peaks. The fundamental study of SERS tags is in urgent need of enhancement. In-depth research on the interaction mechanism between SERS tags and multicomponents is significant. In addition, the development of Raman reporters with different Raman characteristic peaks for multicomponent detection is eagerly awaited. These innovations of SERS tags will definitely help us to design sensitive multiple food contamination detection strategies.
(2) The development of microfluidic technology opens up a new era for SERS detection. A robust microfluidic–SERS integrated platform has great opportunity to automate the detection process. SERS tags injected into microfluidic chips can achieve the automatic separation of multicomponents and the enhancement of Raman signals. Such an integrated platform is expected to achieve batch detection, which will make an important guarantee for food safety.
(3) Considering that food contaminants are relatively complicated, the construction of a SERS-based artificial intelligence database of food contaminant samples is also a burgeoning topic, and future studies are expected to achieve highly sensitive multicomponent detection with less time wasted and less cost.
(4) However, the detection of multiple food contaminants is limited in the laboratory. Thus, SERS-based multicomponent detection in the food industry or in real life needs further research, and great efforts should be made to enhance the testing speed.
(5) In addition to the currently commonly used noble metal SERS substrate, other emerging materials can also exhibit great potential as new generation SERS sensors including MOFs, semiconductors, and perovskites. Future work can rely on several nanomaterials with superior photocatalysis capability and enhance reusability, which are promising in the recycled detection of food multiple contaminants.

Thus, there are enormous challenges and opportunities in exploring the potential multicomponent detection strategies of SERS and their practical utilization in multiple food contaminant control. Upon thoroughly overcoming these challenges, it is highly anticipated that in situ, sensitive and practical multiple contaminant simultaneous determination will be achieved and become a reality for establishing a better future.

## Figures and Tables

**Table 1:** Multiple food contaminants in different matrices simultaneously detected by SERS.

Analytes	Matrices	Sample Pre-Treatment	Substrates	LOD	Detection Ranged	Refs.
Bacteria						
*Escherichia coli*, *staphylococcus aureus*	Water, milk	No pre-treatment	AuNPs were modified by antibodies	10 cfu/mL, 25 cfu/mL	20–50,000 cfu/mL 60–200,000 cfu/mL	[54]
*Listeria monocytogenes*, *salmonella typhimurium*,	Milk	LFIA strip separation	Antibodies modified AuNPs	75 cfu/mL, 75 cfu/mL	10^2^–10^7^ cfu/mL 10^2^–10^7^ cfu/mL	[73]
*Salmonella enterica*, *escherichia coli*, *listeria monocytogenes*	Apple, juice	Bar-chart spin chip separation	PtNP-mediated magnetic DNA aptamer sensors	10 cfu/mL	10–800 cfu/mL 10^2^–10^8^ cfu/mL 10^2^–10^7^ cfu/mL	[80]
*Escherichia coli*, *staphylococcus aureus*	Blood	No pre-treatment	Graphene– AgNPs–Silicon Sandwich SERS Chip	10^6^ cfu/mL		[81]
*Escherichia coli*, *staphylococcus aureus*	Standard solution	Magnetic separation	Vancomycin modified Fe_3_O_4_@AuNPs	20 cfu/mL, 50 cfu/mL	50–10^5^ cfu/mL 20–10^5^ cfu/mL	[65]
*S. typhimurium*, *s. aureus*	Pork	Magnetic separation	Fe_3_O_4_@AuNPs	15 cfu/mL, 35 cfu/mL	10^2^–10^7^ cfu/mL	[49]
*E. coli O157:H7*, *s. typhimurium*	Tap water, cucumber, chicken	No pre-treatment	Aptamers modified AuNRs	5 cfu/mL, 8 cfu/mL	10–10^6^ cfu/mL	[82]
*Vibrio parahaemolyticus*, *salmonella typhimurium*	Seafood	No pre-treatment	Au-PDMS film	18 cfu/mL, 27 cfu/mL	18–1.8 × 10^5^ cfu/mL 27–2.7 × 10^5^ cfu/mL	[67]
Pesticides						
Thiram, malachite green, methyl parathion	Cucumber, green pepper	No pre-treatment	Au@void@Au nanorattles micropipettes	8 nM, 8 nM, 1.5 μM		[83]
Thiram, methyl parathion, malachite green	Apple, cucumber, grape	No pre-treatment	3D PDMS nano-tentacle array coated with AuNPs	1.6 ng/cm^2^	10^−7^–10^−11^ M	[84]
Thiram, methamidophos	Apple	Ethanol extraction	Au@Ag core–shell nanorods	4.6 × 10^−7^ M, 4.4 × 10^−4^ M	4.6 × 10^−7^–3.3 × 10^−4^ M 4.4 × 10^−4^–7 × 10^−3^ M	[85]
Acephate, cypermethrin, tsumacide	Apple	Press and peel off	Au/dragonfly wing substrate	10^−3^ ng/cm^2^, 10^−3^ ng/cm^2^, 10^−4^ ng/cm^2^	10^−3^–10^2^ ng/cm^2^ 10^−3^–10 ng/cm^2^ 10^−4^–10 ng/cm^2^	[86]
Thiram, malachite green	Lake water	No pre-treatment	3D AgNPs /carbon fiber cloth substrate	0.1 ppm	0.1–5 ppm	[87]
Cypermethrin, esfenvalerate	Tap water, river water, milk	SERS-based immunochromatographic assay	Test line with gold nanoparticles	2.3 × 10^−4^ ng/mL, 2.6 × 10^−5^ ng/mL	10^−5^–100 ng/mL	[88]
Cypermethrin, esfenvalerate	Wheat	Molecularly imprinted polymer extraction	AuNPs	2.3 × 10^−4^ ng/mL, 2.6 × 10^−5^ ng/mL	0.05–1 mg/kg	[89]
Thiram, thiabendazole	Apple	Surface magnetic solid-phase extraction	AgNPs- Fe_3_O_4_/Graphene	0.48 ng/cm^2^, 40 ng/cm^2^	0.48 ng/cm^2^–48 μg/cm^2^ 40 ng/cm^2^–40 μg/cm^2^	[90]
Thiacloprid, profenofos, oxamyl	Peach	No pre-treatment	Au@Ag	0.1 mg/L, 0.01 mg/L, 0.01 mg/L	0.1–100 mg/L 0.01–100 mg/L	[91]
Thiram, methyl parathion	Eggplant, Chinese cabbage, grape, strawberry	Ethanol and methanol solutions extraction	MoS_2_/Ag	1.3 × 10^−6^ mg/mL, 1.8 × 10^−6^ mg/mL		[23]
Acetamiprid, 2,4-D	Tea	Solid-phase extraction	Au@Ag	2.63 × 10^−5^ μg/g, 4.15 × 10^−5^ μg/g	1.0 × 10^−4^– 1.0 × 10^3^ μg/g	[92]
Thiram, thiabendazole	Apple, tomato, pear	No pre-treatment	AgNRs array	0.041 ng/cm^2^, 0.79 ng/cm^2^		[34]
Acetamiprid, chlorpyrifos, carbendazim	Apple	Acetone extraction	AgNPs	0.0054 mg/kg, 0.064 mg/kg, 0.014 mg/kg	0.052–1.31 mg/kg 0.61–1.05 mg/kg 0.091–1.35 mg/kg	[93]
Dimethoate, thiuram	Water	Paper separation	AuNPs and ZnONPs	54.57 μg/L, 19.16 μg/L	100–1000 μg/L	[22]
Veterinary drugs						
Sulfamerazine, sulfamethazine, sulfamethoxazole	Pock	No pre-treatment	AuNPs	10 ppb	10 ng/mL–5 μg/mL	[94]
Malachite green, crystal violet	Fish	Acetonitrile extraction	AgNRs	1 ppb		[95]
Food adulterants						
Thiocyanate ion, melamine	Milk, milk powder	Protein precipitation, supernatant extraction	AgNPs	2.00 mg/L, 0.01 mg/L	2.00–190.4 mg/L 0.02–4.8 mg/L	[96]
Prednisone, prednisone acetate, prednisolone, hydrocortisone, hydrocortisone acetate, dexamethasone	Dietary supplements	TLC separation	AgNPs	4 µg, 4 µg, 4 µg, 6 µg, 6 µg, 4 µg	4–10 µg	[97]
Mycotoxins						
Aflatoxin B1, zearalenone, ochratoxin A	Foodstuff	No pre-treatment	AuNPs were modified with 5,5-dithiobis	0.061–0.066 g/kg, 0.53–0.57 g/kg, 0.26–0.29 g/kg		[98]
Aflatoxin B1, Deoxynivalenol, Zearalenone	Maize	Dissolved by methanol	AuNPs-PDMS@AAO template	1.8 ng/mL, 47.7 ng/mL, 24.8 ng/mL	0.005–1 μg/mL 0.1–50 μg/mL 0.05–10 μg/mL	[99]
Aflatoxin B1, zearalenone, fumonisin B1, deoxynivalenol, ochratoxin A, T-2 toxin	Maize	LFIA separation	Au@Ag	0.96 pg/mL, 6.2 pg/mL, 0.26 ng/mL, 0.11 ng/mL, 15.7 pg/mL, 8.6 pg/mL	0.0014–0.33 ng/mL 0.015–3.7 ng/mL 0.41–100 ng/mL 0.14–33.3 ng/mL 0.027–6.7 ng/mL 0.014–3.3 ng/mL	[72]

## Data Availability

Not applicable.
